# Saving Little Lives Minimum Care Package Interventions in 290 Public Health Facilities in Ethiopia: Protocol for a Non-Randomized Stepped-Wedge Cluster Implementation Trial

**DOI:** 10.3390/children13020187

**Published:** 2026-01-29

**Authors:** Abiy Seifu Estifanos, Abebe Gebremaraim Gobezayehu, Mekdes Shifeta Argaw, Araya Abrha Medhanyie, Damen Hailemariam, Bezaye Nigussie Kassahun, Selamawit Asfaw Beyene, Henok Tadele, Lamesgin Alamineh Endalamaw, Abebech Demissie Aredo, Znabu Hadush Kahsay, Kehabtimer Shiferaw Kotiso, Akalewold Alemayehu, Mulusew Lijalem Belew, Amanuel Hadgu Berhe, Simret Niguse Weldebirhan, Asrat Dimtse, Mesay Hailu Dangisso, Samson Yohannes Amare, Yayeh Negash, Abrham Tariku, John Cramer, Siren Rettedal, Abebe Bekele, Fisseha Ashebir Gebregizabher, Selamawit Mengesha Bilal, Meseret Zelalem Tadesse, Dereje Duguma

**Affiliations:** 1 Center for Implementation Sciences (CIS) in Health, School of Public Health, Addis Ababa University, Addis Ababa P.O. Box 1176, Ethiopiaabebech.demissie@aau.edu.et (A.D.A.); 2 Department of Reproductive, Family and Population Health, School of Public Health, Addis Ababa University, Addis Ababa P.O. Box 1176, Ethiopia; 3 Emory University Ethiopia Office, Addis Ababa P.O. Box 9086, Ethiopialijmulusewb@gmail.com (M.L.B.); 4 Department of Pediatrics and Child Health School of Medicine, College of Medicine and Health Sciences, Hawassa University, Hawassa P.O. Box 1560, Ethiopia; 5 School of Public Health, College of Health Sciences, Mekelle University, Mekelle P.O. Box 1871, Ethiopia; araya.medhanyie@mu.edu.et (A.A.M.);; 6 Department of Health Systems and Health Policy, School of Public Health, Addis Ababa University, Addis Ababa P.O. Box 1176, Ethiopia; 7 Department of Pediatrics and Child Health, School of Medicine, College of Health Sciences, Addis Ababa University, Addis Ababa P.O. Box 1176, Ethiopia; 8 Ethiopian Pediatrics Society, Addis Ababa P.O. Box 14205, Ethiopia; 9 School of Public Health, College of Medicine and Health Sciences, Hawassa University, Hawassa P.O. Box 1560, Ethiopia; akemastyle@hu.edu.et; 10 Department of Pediatrics and Child Health, College of Health Sciences, Mekelle University, Mekelle P.O. Box 231, Ethiopia; 11 Ethiopian Public Health Institute, Addis Ababa P.O. Box 1242, Ethiopia; 12 School of Computing, Ethiopian Institute of Technology-Mekelle, Mekelle University, Mekelle P.O. Box 1871, Tigray, Ethiopia; samson.yohannes@mu.edu.et; 13 United Nations Children’s Fund, Addis Ababa P.O. Box 1169, Ethiopia; 14 Ministry of Health, Riyadh P.O. Box 21217, Ethiopia; 15 Implementation Science in Global Reproductive Health (Ethiopia), Emory University, Atlanta, GA 30322, USA; 16 Faculty of Health Sciences, University of Stavanger, 4068 Stavanger, Norway; 17 Department of Paediatrics, Stavanger University Hospital, 4068 Stavanger, Norway; 18 Oromia Regional Bureau, Addis Ababa P.O. Box 24341, Ethiopia; 19 Tigray Regional Health Bureau, Mekelle P.O. Box 07, Ethiopia; 20 Sidama Regional Health Bureau, Hawassa P.O. Box 149, Ethiopia

**Keywords:** neonatal mortality, prematurity, low birth weight, birth asphyxia, neonatal infections

## Abstract

Background: Neonatal mortality remains a significant public health challenge in Ethiopia. Despite efforts to implement key evidence-based interventions, their coverage and utilization remain low. The Saving Little Lives (SLL) program aims to scale-up a Minimum Care Package (MCP) of synergistic, life-saving interventions for all liveborn neonates, with a focus on preterm and low birth weight (LBW) infants, across 290 hospitals in Ethiopia (206 primary, 69 general, and 15 referral hospitals), representing 82% of all hospitals in the country at the time of the study, and evaluate the impact on neonatal mortality. Methods: A non-randomized stepped-wedge trial will be conducted to evaluate the impact of implementing the SLL MCP interventions. Quantitative evaluation data will be collected from 36 primary hospitals, selected from 206 primary hospitals across four regions, receiving the interventions. An independent evaluation research assistant will be deployed in each of the hospitals to collect data using Open Data Kit (ODK) through interviewing mothers before discharge, on the 29th day of life if discharged, and reviewing medical records. A mixed-method, cross-sectional formative assessment will be conducted prior to implementation, employing quantitative facility assessment and qualitative interviews with mothers, healthcare providers, and facility managers. This will be followed by continuous program learning assessment once implementation begins. Descriptive data will be presented using numbers, percentages, tables, and graphs. Regression modeling and generalized estimating equations (GEEs) will be used to estimate the impact of the SLL MCP interventions. Qualitative data will be gathered through in-depth interviews, digitally recorded, transcribed, and thematically analyzed using ATLAS.ti Version 7.5 software to assess facility readiness, barriers, and enablers of implementing the SLL MCP interventions. Expected Outcome: We hypothesize that achieving 80% coverage of the SLL MCP interventions among eligible neonates will result in a 35% reduction in neonatal mortality at implementation facilities.

## 1. Introduction

Despite significant global progress in reducing under-five child mortality over the past two decades, neonatal deaths have remained steady, accounting for 47% of total under-five deaths in 2022. Of the 2.3 million neonatal deaths every year, 46% occur in sub-Saharan Africa [1]. With an estimated 33 neonatal deaths per 1000 live births in 2019, Ethiopia is far from meeting United Nations Sustainable Development Goal (SDG) 3.2.2 of achieving less than 12 neonatal deaths per 1000 live births [1]. Complications due to prematurity, infection, and birth asphyxia contribute to more than 74% of neonatal deaths [2], and about 80% of neonatal deaths occur among preterm (born before 37 weeks of gestation) or low birth weight (born with less than 2500 g weight) newborns [3]. Among premature neonates, deaths are mostly due to respiratory distress syndrome (RDS) (45%), infection (30%), and birth asphyxia (13%) [4]. Hence, evidence for strategies to effectively identify and manage drivers of neonatal morbidity and mortality are critically needed to promote neonatal survival.

A considerable number of contributors to neonatal mortality are preventable through the effective implementation of existing evidence-based interventions. Investments in ensuring quality maternity and newborn care at hospitals, making infrastructure and device bundles available in Neonatal Intensive Care Units (NICUs), and implementing infection prevention measures are crucial to accelerating progress toward SDG goals for ending preventable deaths globally [5]. The World Health Organization (WHO) recommends continuous positive airway pressure (CPAP) to manage respiratory distress syndrome (RDS) in preterm newborns [6]. Infection prevention and management of sepsis using antibiotics are also key interventions to prevent neonatal deaths caused by infection [6,7]. A significant proportion of deaths due to birth asphyxia can be avoided through timely and efficient bag-mask ventilation after birth [8]. Other evidence-based interventions include antenatal corticosteroids for pregnant mothers at imminent risk of preterm birth [9,10], and Kangaroo Mother Care (KMC) for preterm and low birth weight (LBW) newborns [6]. In addition, improving feeding practices among preterm and LBW newborns can reduce the risk of neonatal death, up to 45% of which is attributed to malnutrition [11].

The Ethiopian Newborn and Child Survival and Development Strategy prioritizes antenatal corticosteroids for preterm labor, neonatal resuscitation and respiratory support, KMC, antibiotics for neonatal sepsis, and early initiation and exclusive breastfeeding as key interventions to improve neonatal survival [7]. Furthermore, Ethiopia has been making substantial national investments to improve neonatal care across the health system, as articulated in strategic documents, strategies, and guidelines [12,13]. These strategy documents provide guidance for achieving a substantial reduction in neonatal mortality (to 21 deaths per 1000 live births) by 2025 [14]. However, Ethiopia remains among the five countries with the highest neonatal mortality rates [1]. More importantly, the coverage of key interventions remains low, resulting in high neonatal mortality rates.

Building on the lessons learned from the successful scale-up of KMC implementation research conducted in collaboration with the WHO to scale up KMC in four major regions of the country between 2016 and 2019 [15], the SLL MCP program aims to scale up lifesaving interventions for eligible liveborn infants, with a focus on preterm and LBW newborns. We hypothesized that the estimated mortality reduction target will be met by achieving 80% coverage of an evidence-based bundle of interventions for eligible neonates during childbirth, in the NICU, and in the KMC unit, with a focus on preterm and LBW newborns, in 290 hospitals across four regions in Ethiopia over a three-year implementation period. Given that the hospitals covered by the SLL MCP program represent 82% of the total 353 hospitals operating in the country [16], and in accordance with the government’s strategy for comprehensive newborn care and leveraging the existing structures of the national and regional health system, led by the government, the objective of this project is to reduce neonatal mortality by 35% in the implementation centers, measuring the coverage and impact of implementing the SLL MCP interventions on neonatal mortality.

## 2. Materials and Methods

### 2.1. Setting

Ethiopia is a federal republic. At the time of protocol development, the country was administratively divided into nine regions (the highest level of administrative division), 66 zones (intermediate administrative division), and 670 woredas (the third level and basic unit of local government, responsible for service delivery and governance at the community level). Ethiopia has a three-tier service delivery system. The primary level of care consists of primary hospitals, health centers, and health posts, each serving a population of 60,000–100,000. The secondary level of care comprises general hospitals, each serving a catchment population of 1,000,000–1,500,000. The tertiary level of care is composed of referral and specialized hospitals, each serving a catchment population of 3,500,000–5,000,000 [12].

The SLL MCP interventions will be implemented in 290 hospitals across four regions in Ethiopia: Oromia, Amhara, Tigray, and Southern Nations, Nationalities, and People (SNNP). These four regions are home to more than 85% of the country’s population (estimated 84.6 million). According to the Central Statistics Agency’s 2019 population projection, Ethiopia’s total population was estimated at 98.7 million [17]. The latest UNESCO report indicates that the literacy rate among individuals aged 15 and above in Ethiopia is 52% (59% for males and 44% for females). Table 1 provides a summary of the estimated population, annual total births, annual <2500 g births, annual <2000 g births, and annual preterm births in the catchment woredas of SLL-supported hospitals.

### 2.2. Study Design

We will conduct a non-randomized stepped-wedge implementation study from 1 June 2021 to 30 May 2024 (Table 2).

The implementation of the SLL MCP interventions will be carried out in three phases, with SLL-supported hospitals intentionally selected in these phases. In phase one, intervention facilities adjacent to the scale-up KMC implementation research sites will be chosen in collaboration with regional health bureaus to maximize skill transfer from facilities involved in the KMC scale-up implementation research to new SLL facilities [15]. In phases two and three, sites will be enrolled based on their proximity to phase one hospitals to facilitate the diffusion of skills and experience. Eventually, all targeted facilities will receive the SLL MCP interventions. Because the intervention timing is sequential and evaluation data will be collected throughout the project across all evaluation sites, each facility will participate in both the intervention and comparison periods. This approach is assumed to enhance the statistical power to assess the interventions’ effect.

To evaluate the coverage and impact of the SLL MCP interventions, 36 primary hospitals will be randomly selected from the total 206 primary hospitals across the four regions receiving the SLL MCP interventions over the three implementation cycles. General and referral hospitals are excluded from the sample because the project aims to evaluate the impact of the SLL MCP interventions at the lowest level of hospitals in the health system where the interventions are most needed. After the primary hospitals that will receive the interventions in the first, second, and third phases are identified in collaboration with the regional health bureaus, the 36 evaluation sites will be randomly selected, with 12 from each implementation cycle and 9 from each of the four regions.

Additionally, the study will conduct a mixed-method cross-sectional formative assessment using qualitative and quantitative data collection tools from the hospitals at the beginning of each implementation phase. The assessment aims to assess the readiness of the facilities to implement the SLL MCP interventions and identify contextual barriers and facilitators to the implementation of the SLL MCP interventions. To guide the formative assessment, we adapted the Consolidated Framework for Implementation Research (CFIR).

During the implementation of the SLL MCP interventions, ongoing assessment of program learning will be conducted, following the CFIR domains, to generate learning through the continuous collection and analysis of qualitative data, which will be used throughout the study to improve implementation performance.

Furthermore, the project will conduct incremental cost analysis for implementing the SLL MCP interventions at the evaluation hospitals. The proposed approach focuses on estimating the cost effectiveness (one-time fixed costs and recurring costs at each phase of scale-up) of the SLL MCP project. Intervention activities will be costed at the (a) project level and (b) health facility level. A micro-costing approach will be adopted to include a bottom–up construction of costs associated with integrating the SLL MCP interventions into the current practice. With such an ingredient costing approach, costs will be determined whereby actions to be taken under scale up of the SLL MCP interventions are listed, additional specific resources needed to implement the interventions are described, and prices are assigned for all the resources based on opportunity costs, as well as based on financial records and from interviews with program managers.

Table 3 summarizes the study components in this project, the type of statistical evaluation, the target population, and the data collection in each case.

### 2.3. Study Participants

The SLL MCP interventions will target all mother–neonate dyads, but will most benefit preterm and LBW newborns who are at highest risk of dying in the first month of life. To measure the impact of the SLL MCP interventions, all live births and newborns who receive care at the 36 evaluation primary hospitals are eligible for inclusion in the study. Mothers who have stillbirths will be excluded as their stillborn is not eligible to receive the SLL MCP interventions, most of which is provided starting immediately after birth.

In addition, the study will include health facility administrators, healthcare providers, mothers giving birth in hospitals and their families, and mothers and their families whose newborns are admitted to NICU and KMC units, with the goal of gathering information on the care experience of administrators and providers and on the care received by mothers and their families, especially those with premature and low birth weight newborns.

### 2.4. SLL MCP Intervention Packages

The SLL MCP interventions aim to enhance care quality for all neonates, particularly focusing on the most vulnerable small and preterm newborns, by strengthening hospitals’ ability to manage these vulnerable newborns. The SLL MCP interventions will implement a low-dose high-frequency clinical mentorship model [18] to build the competency and confidence of healthcare providers in labor and delivery, NICU, and KMC units in care provision for all neonates, with a special focus on small and preterm babies who are at risk of a range of complications.

**Figure 1:**
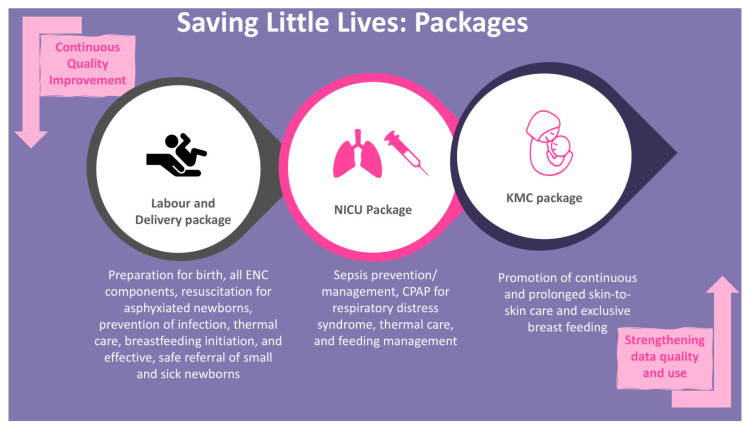
Saving Little Lives intervention packages. NICU: Neonatal Intensive Care Unit, KMC: Kangaroo Mother Care, ENC: Essential Newborn Care, CPAP: Continuous Positive Airway Pressure.

To achieve the project’s set goal of neonatal mortality reduction, the SLL MCP interventions address three point-of-care areas: (i) labor and delivery, (ii) NICU, and (iii) KMC units. The SLL MCP package in the labor and delivery unit includes preparation for birth (confirming essential supplies such as magnesium sulphate, antibiotics, sanitizers are available, birth companion identified and prepared, delivery room is cleaned and warmed, and delivery kits prepared), essential newborn care components including resuscitation for asphyxiated newborns, CPAP at labor and delivery, prevention of infection, thermal care, early initiation and exclusive breastfeeding, and safe referral (provide pre-referral treatment, inform family on referral, prepare referral note, identify provider to accompany the newborn during referral, and arrange emergency transport service) of small and sick neonates. The SLL MCP intervention package in the NICU includes care recommendations for the level of the facility, such as management of bacterial infections including sepsis, CPAP for RDS, thermal care, and feeding management. The KMC care package consists of exclusive breastfeeding and skin-to-skin care for at least 8 h per day in line with WHO recommendations (See Figure 1).

### 2.5. Implementation Strategy

**Figure 2:**
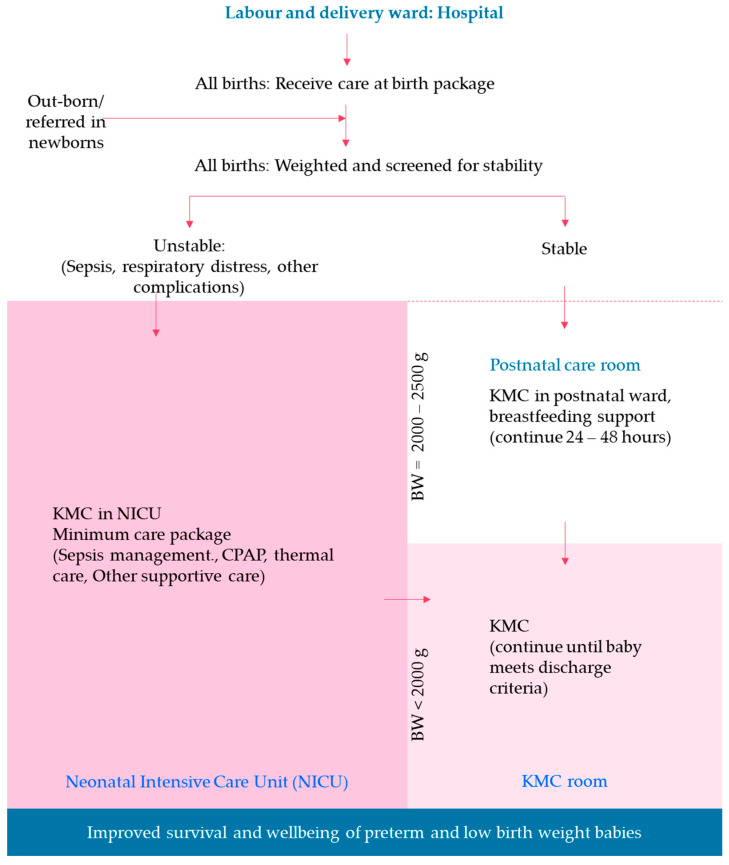
Implementation strategy for Saving Little Lives Minimum Care package in Ethiopia. KMC: Kangaroo Mother Care, NICU: Neonatal Intensive Care Unit, CPAP: Continuous Positive Airway Pressure.

At the hospital level (Figure 2), all newborns at SLL MCP implementation hospitals will receive care immediately after birth, which includes preparing for and providing clean and safe childbirth care. During the intrapartum period, maternal and fetal statuses will be monitored, and complications will be managed, including resuscitation for asphyxiated newborns, prevention of infection, thermal care, and breastfeeding initiation. Maternal and neonatal care will continue at the postnatal care unit for 24–48 h until the mother is discharged.

Newborns will be screened for any complications. Small and/or sick newborns in need of additional and supportive care will be linked with or referred to an appropriate care facility or unit. Newborns who have complications such as RDS, sepsis, or other complications will be admitted to the NICU and receive appropriate care. Newborns who weigh less than 2000 g at birth and who are stable will be transferred to the KMC unit for continuous KMC, which includes prolonged skin-to-skin care and exclusive breastfeeding until they meet the discharge criteria (Figure 1).

Guided by the Institute for Healthcare Improvement (IHI) framework for innovation spread, the national quality strategy, and WHO maternal and newborn health quality standards, we will develop/adapt and use care at birth, NICU, and KMC quality standards, verification criteria, and assessment tools by aligning WHO recommendations with national guidelines and protocols to continuously assess the input, process, and output performance of the implementation facilities and sites. We will adapt Plan–Do–Study–Act (PDSA) continuous quality improvement cycles to use the data from quality assessments and root cause analysis using tools such as fish bone diagrams and program implementation learning to refine the implementation and improve the performance of SLL MCP implementation.

To select the hospitals that will be enrolled into the program, the regional health bureaus will use criteria including, first, for the first phase SLL MCP implementation facilities, their proximity to the previous KMC project implementation research facilities to facilitate the systematic transfer of experiences and skills gained from KMC implementation. For the second and third phase facilities, respective regions shall follow a similar approach, ensuring the proximity of the hospitals to the first phase hospitals. Second, the minimum readiness of the facilities to implement the SLL MCP interventions in terms of their healthcare providers, infrastructure, and leadership engagement. Third, the physical accessibility of the facilities to the mentoring hospital for frequent mentorship and technical support. Finally, alignment of the facilities with the government catchment/network of care facility approach to strengthen the existing government referral and linkage system.

Clinical mentors will be recruited from higher-level hospitals and receive training from trainers on the SLL MCP packages. The mentors’ competence will be evaluated using structure knowledge and skills assessment tools by senior neonatologists, obstetricians, and genealogists, who will train them, and those who acquire the necessary skills will participate in the mentorship program. Clinical mentors will similarly evaluate the knowledge and skills of physicians, midwives, and NICU nurses using structured knowledge and skills assessment tools tailored to the care the mentees provide, as well as the conditions of the space, infrastructure, and equipment in labor and delivery, NICU, and KMC units. They will then collaborate with the staff of the units and hospital leadership to develop a plan to address any gaps. The clinical mentors will reorganize the units based on these gaps to enhance the care process and provide space for neonate–mother dyads, install selected devices and equipment from hospital resources or acquired through the SLL MCP interventions, and offer ongoing, demand-driven, hands-on, catchment-based clinical mentorship and training at SLL MCP implementation facilities to improve care in labor and delivery, NICU, and KMC units. Clinical mentorship guides and tools (including mentorship process and mentees’ competency assessment tools) tailored to the care for all neonates, with a special focus on preterm and LBW newborns, will be developed in consultation with the Ministry of Health, regional health bureaus, UNICEF, the WHO, the Ethiopian Pediatric Society (EPS), the Ethiopian Society of Obstetricians and Gynecologists (ESOG), and the Ethiopia Midwives Association (EmWA). The mentorship data will be analyzed and used to inform refinement of the subsequent mentorship support. Hospitals will conduct quarterly catchment implementation learning meetings to standardize care, review performance, cross-learn experiences among facilities, and refresh their knowledge on key SLL MCP intervention areas.

### 2.6. Sample Size Estimation

While the SLL MCP interventions will be implemented in 290 hospitals consisting of primary, general, and referral hospitals, only live births and newborns treated in primary hospitals will be included and followed until their 29th day of life to estimate the impact of the intervention on neonatal mortality.

To estimate the impact of the SLL MCP interventions on neonatal mortality, the sample size required to detect a 35% reduction in neonatal mortality after implementation of the SLL MCP interventions is calculated using a sample size calculator for a stepped-wedge design. Assuming that a woreda will have a minimum population of 30,000, and with a conservative estimate of a crude birth rate of 20 births per 1000 population, there will be a minimum of 600 births per cluster (woreda) in a year. We estimated the sample size using an online sample size calculator for a stepped-wedge design (https://clusterrcts.shinyapps.io/rshinyapp/, Accessed on: 13 May 2021), considering a cross-sectional sampling structure, a discrete time decay correlation structure, 3 sequences, 0.02 ICC, and 0.8 cluster autocorrelation. The neonatal mortality under control and intervention conditions was considered to be 33 and 22 per 1000 live births, respectively, with 80% power and a 5% level of significance. The sample size required to detect a 35% reduction in neonatal mortality in the implementation sites is 12 clusters per sequence (36 clusters overall). At each phase of SLL MCP implementation, an estimated 21,600 newborns across the four implementation regions will be screened to assess neonatal outcomes.

An SLL MCP cluster will be a primary hospital that covers a woreda with an estimated minimum population of 30,000. Typically, a woreda has one primary hospital with referral linkage to a general and a referral hospital. In this evaluation design, we will progressively roll out the interventions by cluster. From the 286 woredas, the impact of the SLL MCP interventions will be measured in 36 selected woredas, hereafter referred to as evaluation clusters (9 clusters per region).

For formative assessment and program learning, qualitative data will be collected through interviews with purposively selected preterm newborns’ mothers, healthcare providers, health facility managers, mentors, and mentees involved in obstetric and newborn care, the NICU, and KMC unit at the intervention facilities. While facility assessment will be carried out in the 290 hospitals, the qualitative assessment of participants and the number required for formative assessments and program learning will be determined case by case across each phase depending on the nature of the implementation process and the hospital setting.

### 2.7. Data Management

#### 2.7.1. Data Collection Procedure and Tool

Quantitative impact evaluation data will be collected from 36 evaluation sites. Evaluation research assistants (ERAs) with experience working at the NICU will be trained and deployed at the selected evaluation sites to generate data for all live births. The ERAs will enroll and interview mothers at labor and delivery, NICU, and KMC units. In addition, they will extract data from patient charts and registers and make phone calls to parents of each baby on the 29th day of life to follow up on the survival status of the neonate.

To complement the evaluation data generated by the ERAs and understand the patterns and trends of neonatal mortality at the hospitals, before and after SLL MCP implementation, data will also be extracted from the District Health Information System (DHIS2) and logbooks of SLL-supported hospitals. A data extraction tool covering key variables from labor and delivery as well as NICU logbooks will be developed and used.

The mixed-method formative assessments will generate qualitative and quantitative data from managers, healthcare providers, and mothers who give birth at the hospitals and mothers with a newborn/s admitted to NICU and KMC units through in-depth interviews and observations. To guide the development of formative assessments and continuous program learning data collection tools, we adapted the CFIR [19,20]. Facility readiness will be assessed using the World Health Organization’s Service Availability and Readiness Assessment tool. Guided by the CFIR, we will develop in-depth interview guides tailored to the study participant groups.

The process and outcomes of implementing the SLL MCP interventions will be continuously monitored after the interventions are introduced. Monitoring will be accomplished using the government routine data from DHIS2 and independent data that the project evaluation research assistants and program learning team will collect from a sub-sample of users, care providers, and health facilities.

Data shall be collected by hospital-based evaluation research assistants who will be located at each of the 36 primary hospitals and supervised by evaluation coordinators based at SLL university partners with an oversight of the investigators. The data collectors and evaluation coordinators will receive training on the SLL MCP project objective, standard operating procedure, tools, and consent processes. They will also receive onsite mentorship and support from investigators until they are proficient in data collection, quality control, and management.

Qualitative data will be collected by the formative assessment and program learning team with experience in qualitative data collection who will receive additional training on qualitative data collection. The interviewers will purposively select mothers and family members who can provide rich information on their experience of care from the postnatal care ward, NICU, and KMC units. They will follow similar approaches to identify and interview healthcare providers and facility administrators engaged in the provision of care at birth, in the NICU, and in the KMC unit at the hospitals.

Cost data including personnel; drugs and supplies; space, equipment, and furnishing items; as well as administrative and other overheads will be collected. A structured cost data collection tool will be used to extract these cost data elements.

#### 2.7.2. Data Sources

Quantitative data shall be collected electronically using Open Data Kit (ODK). ODK was chosen over other platforms with similar functions because it is open source, has good support for offline data collection, and it supports several local languages. Given the high rate of network interruption, this tool can be set up offline, and data can also be collected in remote areas without much difficulty. Moreover, it is user friendly and finding evaluation research assistants who are familiar with the software is much easier.

Data will be collected from three major sources. There will be continuous data collection of all live births, NICU, and KMC admissions to assess the quality and coverage of the SLL MCP interventions and outcomes at different time intervals based on the stepped-wedge design.

Routine data collected by the health system DHIS2 will also be used to measure intervention effective coverage. DHIS2 is an electronic HMIS data reporting platform that is being scaled-up in Ethiopia. Currently, all hospitals in Ethiopia report their aggregate routine data using DHIS2. DHIS2 captures relevant coverage and impact indicators on neonates treated at different health facilities in the SLL MCP scale-up woredas. Since SLL MCP is a government owned and led initiative, routine monitoring of the implementation performance of the project will be performed through the existing government DHIS2 platform. However, the data from DHIS2 have gaps in quality and not all of the SLL MCP intervention outcome indicators are routinely reported. SLL will work with UNICEF, the Ministry of Health, regional health bureaus, and health facilities to include additional indicators to measure effective coverage of the intervention package, improve the quality of data captured and reported through DHIS2, monitor each of the interventions in the SLL MCP packages during the scale-up period, and ensure the monitoring system is sustained beyond the project period.

Each region and university partner supporting SLL MCP implementation in the region will have full ownership of the data generated at their region and lead and coordinate quality data collection in the region. In the online ODK-based data management platform, separate projects shall be created for each region. Data can then be easily merged, as all sites will have the same tools and design. The tools will be developed, tested, and deployed after the proper training of evaluation research assistants. Pre-testing of tools and appraisal of the readiness of the data collection platform will be coordinated centrally. All eligible mothers will be consecutively interviewed about their pregnancy history, experiences, antenatal care, delivery care, neonatal care, birth outcome, and neonatal outcomes of their last live birth.

Qualitative data from in-depth interviews (IDIs) will be digitally recorded and immediately translated, transcribed, and submitted electronically using Microsoft Word format. Field notes shall also be collected and submitted electronically immediately from the respective sites. Sites shall safely store the data and send it to central data management within a month.

Intervention activities will be costed at two levels: program level (cost of project coordination and program management, procurement and installation of equipment and education materials, and training of project coordinators and healthcare providers), and health system level (value of healthcare providers’ time, cost of additional hired providers, cost of additional equipment and supplies, cost of awareness creation activities among healthcare providers and communities).

#### 2.7.3. Data Quality

Quality checks will be performed from the outset so that corrective measures can be taken promptly. Data quality measures shall be designed during the development of ODK forms in such a way that outliers, data type checks, and data validations are built into the electronic data collection tool design. After being submitted to the server, the data shall be checked for quality immediately and feedback shall be given to the evaluation research assistants in real time. All data cleaning tasks shall be performed using a do file in STATA software by keeping the original dataset in place, ensuring a quality data pipeline.

#### 2.7.4. Data Analysis

The quantitative data will be cleaned and analyzed using STATA software version 14 or higher (StataCorp LLC, College Station, TX, USA). Descriptive analysis of the primary and secondary outcomes and independent variables will include data presentation and summarization using percentages (frequency distribution), tables, and graphs. To measure the impact of the SLL MCP interventions on neonatal mortality, regression modeling will be employed. Potential confounding variables such as maternal and neonatal characteristics, facility type, and region will be controlled during the regression analysis. Generalized estimating equations (GEEs) will be used to address clustering and correlation effects.

Both the formative assessment and continuous program learning will be conducted in three steps. First, an in-depth assessment of facility readiness, enablers, and barriers for provision of quality care for preterm and low birth weight newborns at labor and delivery, NICU, and KMC units will be completed. Then, a preliminary data analysis using a thematic framework analysis method will be completed. Finally, a data analysis review and validation workshop that brings together key stakeholders from the Ministry of Health, regional health bureaus, hospitals, and university research partners will be conducted. In the workshop, the hospitals will be engaged in the review of the qualitative and quantitative data, validate the key findings, and suggest solutions to overcome barriers in their own hospitals.

Incremental cost analysis will be performed to estimate the unit cost for neonatal life saved due to implementation of the SLL MCP interventions in the existing health system. Analyses of collected cost data will be undertaken from the health system’s perspective.

## 3. Discussion

Ethiopia is currently off track in achieving the SDG 3.2.2 target for neonatal mortality. The goal for the country is to reduce the neonatal death rate from 33 per 1000 live births in 2019 to less than 12 per 1000 live births. The proposed evaluation will utilize a non-randomized stepped-wedge design to examine the effect of implementing the SLL MCP interventions in reducing neonatal mortality. Specifically, the study will test the hypothesis that the SLL MCP interventions can reduce neonatal mortality by 35% among inborn neonates at the hospital level by increasing intervention coverage to 80% of eligible neonates, with a focus on preterm or LBW neonates. The uptake and outcomes of the SLL MCP interventions will be recorded at discharge and on the 29th day of life.

The implementation strategy of the SLL MCP project is informed by prior successful and proven experience during the scale up of the KMC project. The implementation of life-saving interventions in the first month of life is challenged by a range of factors related to parents, healthcare providers, health facilities, and the broader health system context of countries. Hence, the implementation of life-saving interventions for preterm and LBW neonates demands countries to invest in increasing the number of staff and enhancing their technical skill, improving health facility infrastructures, and strengthening healthcare leadership [5]. The implementation of large-scale neonatal care interventions and systematically evaluating their effects using empirical data will better inform the maternal and neonatal health programs of the Ministry of Health, regional health bureaus, and other countries in a similar context.

The study will employ a non-randomized stepped-wedge design, enabling real-world implementation research of programs such as SLL MCP to be practically implemented over time and robustly evaluated [21].

### Limitations of the Study

The SLL MCP program is the largest facility-based neonatal care program in Ethiopia, marking a bold and ambitious step in applying implementation sciences to scale up and evaluate a large-scale health system program in the country. Nevertheless, the program’s small budget limited our capacity to plan for adequate resources to address the substantial gaps in health systems, particularly at primary hospitals. Excluding general and referral hospitals from the evaluation denominator could potentially diminish the potential impact of the program, as these hospitals are typically more prepared to implement the SLL MCP interventions than primary hospitals. Moreover, the program does not include interventions at health centers and the community level, missing out on a significant proportion of births at health centers and homes. A randomized stepped-wedge design is not feasible because the main hospitals for the three SLL MCP implementation periods will be selected by the management of the regional health offices, which does not allow for randomization. Given the resource limitations in collecting client cost data, the incremental cost analysis will be restricted to the health system perspective, without accounting for client costs. During the development of this protocol, Ethiopia was in the middle of the COVID-19 pandemic and war in Tigray. Such limitations and unforeseen challenges can pose risks in the achievement of the ambitious goal of reducing neonatal mortality by 35%.

## 4. Conclusions

This protocol outlines a systematic approach to scaling up the SLL MCP interventions across 290 hospitals in Ethiopia and evaluating their impact on neonatal mortality. The study addresses critical gaps in the scale up and evaluation of evidence-based large-scale complex health system interventions. The findings from this study will inform similar maternal and neonatal health programs in Ethiopia and in other low- and middle-income countries sharing a similar context.

## Figures and Tables

**Table 1:** Total expected population and birth categories in SLL MCP implementation sites in Ethiopia, 2019.

Region	Estimated Total Population	Estimated Total Annual Births	Estimated Total Annual <2500 g Births	Estimated Total Annual <2000 g Births	Estimated Total Annual Preterm Births
Tigray	5,541,997	139,455	10,599	4184	13,946
Amhara	22,192,000	342,600	76,057	10,278	34,260
Oromia	38,170,000	528,588	69,245	15,858	52,859
SNNP	10,371,000	267,233	35,008	8017	26,723
Total	76,274997	1,277,876	190,908	38,336	127,788

**Table 2:** A non-randomized stepped-wedge design to evaluate the coverage and impact of the SLL MCP interventions in four regions of Ethiopia, 2021.

Phases	3 Months	9 Months	3 Months	9 Months	3 Months	9 Months
Phase 1	Routine practice	Intervention *				
Phase 2	Routine practice	Routine practice	Routine practice	Intervention		
Phase 3	Routine practice	Routine practice	Routine practice	Routine practice	Routine practice	Intervention
Continuous program learning data collection, analysis, and use to inform intervention refinement and improve program performance						

* Shaded areas in the Table indicate intervention period.

**Table 3:** Summary of the study components, participants, and data sources.

Study Component	Design	Participants	Data Sources
Evaluation of the impact of the SLL MCP interventions	Non-randomized stepped-wedge design	Mother–newborn dyads: all live births who receive care at the 36 evaluation primary hospitals	Independent evaluation research assistants collect data by interviewing mothers and family members, reviewing medical charts and registers
Assess facility readiness, barriers, and facilitators of SLL MCP implementation	A mixed method cross-sectional formative assessment	Health facility managers, healthcare providers, and mothers who give birth at the hospitals and mothers with a (preterm) newborn/s admitted to NICU and KMC units	Facility observation, interviews with facility managers, healthcare providers, and mothers
Document program learning during SLL MCP implementation	Continuous qualitative assessment	Facility managers, healthcare providers, and mothers who give birth at the hospitals and mothers with a (preterm) newborn/s admitted to NICU and KMC units	Interviews with facility managers, healthcare providers, and mothers
Assess the cost effectiveness of the SLL MCP interventions	Cost description and cost analysis on the incremental cost of scaling up the SLL MCP interventions in the hospitals	Project managers, facility managers, and healthcare providers	Review of program financial reports, interview of project managers, facility managers, and healthcare providers

SLL—Saving Little Lives, MCP—Minimum Care Package, KMC—Kangaroo Mother Care, NICU—Neonatal Intensive Care Unit.

## Data Availability

The data for this study consist of quantitative and qualitative data. For the quantitative data, individual participant data will not be made public. Quantitative data will be made available upon request by users. The data request should be submitted to thecis@aau.edu.et along with the planned secondary analysis. The SLL MCP principal investigator group will review the data access request and analysis plan and grant access to users. Qualitative data will not be made available as it will be difficult to anonymize the identity of the study participants.

## Abbreviations

The following abbreviations are used in this manuscript:

CPAP	Continuous Positive Airway Pressure
KMC	Kangaroo Mother Care
LBW	Low Birth Weight
MCP	Minimum Care Package
NICU	Neonatal Intensive Care Unit
RDS	Respiratory Distress Syndrome
SLL	Saving Little Lives
WHO	World Health Organization
